# *C*_3_-symmetric opioid scaffolds are pH-responsive DNA condensation agents

**DOI:** 10.1093/nar/gkw1097

**Published:** 2016-11-29

**Authors:** Natasha McStay, Zara Molphy, Alan Coughlan, Attilio Cafolla, Vickie McKee, Nicholas Gathergood, Andrew Kellett

**Affiliations:** 1 School of Chemical Sciences and National Institute for Cellular Biotechnology, Dublin City University, Glasnevin, Dublin 9, Ireland; 2 School of Physical Sciences, Dublin City University, Glasnevin, Dublin 9, Ireland; 3 Department of Chemistry, Tallinn University of Technology, Akadeemia tee 15, 12618 Tallinn, Estonia

## Abstract

Herein we report the synthesis of tripodal *C*_3_-symmetric opioid scaffolds as high-affinity condensation agents of duplex DNA. Condensation was achieved on both supercoiled and canonical B-DNA structures and identified by agarose electrophoresis, viscosity, turbidity and atomic force microscopy (AFM) measurements. Structurally, the requirement of a *tris*-opioid scaffold for condensation is demonstrated as both di- (*C*_2_-symmetric) and mono-substituted (*C*_1_-symmetric) mesitylene-linked opioid derivatives poorly coordinate dsDNA. Condensation, observed by toroidal and globule AFM aggregation, arises from surface-binding ionic interactions between protonated, cationic, tertiary amine groups on the opioid skeleton and the phosphate nucleic acid backbone. Indeed, by converting the 6-hydroxyl group of *C*_3_-morphine (**MC3**) to methoxy substituents in *C*_3_-heterocodeine (**HC3**) and *C*_3_-oripavine (**OC3**) molecules, dsDNA compaction is retained thus negating the possibility of phosphate—hydroxyl surface-binding. Tripodal opioid condensation was identified as pH dependent and strongly influenced by ionic strength with further evidence of cationic amine-phosphate backbone coordination arising from thermal melting analysis and circular dichroism spectroscopy, with compaction also witnessed on synthetic dsDNA co-polymers poly[d(A-T)_2_] and poly[d(G-C)_2_]. On-chip microfluidic analysis of DNA condensed by *C*_3_-agents provided concentration-dependent protection (inhibition) to site-selective excision by type II restriction enzymes: BamHI, HindIII, SalI and EcoRI, but not to the endonuclease DNase I.

## INTRODUCTION

The search for new synthetic DNA recognition agents is an area of considerable research importance. These agents can be categorized, broadly, into two main areas: (i) those that covalently bind nucleic acids at electron rich sites on the nucleobase (1–3) and (ii) those that bind in non-covalent fashion through hydrogen (H)-bonding, ionic or π-stacking interactions (4–6). Accordingly, non-covalent recognition scaffolds may be further classified to the area of DNA where recognition occurs thus giving rise to groove binders (7–9), intercalators (9–12) (including threading intercalators (13)) and surface-binding compounds (14). Within this last category, surface interacting compounds have been designed, and compared to naturally occurring protein binding domains containing, for example, cationic arginine (arginine fork (15)) or lysine (poly-*L*-lysine (16)) amino acid residues that facilitate their complexation to nucleic acids. Indeed, a commonality between synthetic and naturally occurring surface binders is their ability to efficiently bind electron-rich phosphate oxygen atoms located at the nucleic acid backbone, principally through H-bonding and electrostatic interactions. Furthermore, electrostatic binding by polycationic molecules can directly lead to a collapse in the B-DNA conformation with resultant condensation or aggregation of tertiary DNA helical structures (17). Condensation agents have, in turn, found widespread utility in molecular biology as transfection agents capable of mediating cellular delivery of nucleic acids for ubiquitous therapeutic and bioprocessing applications (18). Thus, an important consideration in the development of new DNA condensation agents is their relative inertness toward chemical reactivity (e.g. artificial nuclease) or secondary interactions (e.g. intercalation) that may interrupt or block efficient transport and incorporation of target nucleic acids into mammalian cells.

While nucleic acid condensation can be achieved through mechanisms of neutral crowding, antisolvent precipitation and liposomal packaging (amongst others), significant attention focuses on the condensation properties of multivalent cationic compounds including cationic polymers. Although DNA condensation has been reviewed by Bloomfield (17) and others including theoretical descriptions of the dynamics of polymer collapse (19,20), it is worth considering some examples of multivalent cations currently accessible to this field. Cationic charge density—essential for the neutralization of anionic phosphate charge and the reorganization of water dipoles adjacent to DNA surfaces—plays a critical role in driving condensation and the majority of cationic agents carry a formal charge of 3+ or higher. Prominent examples here include the naturally occurring polyamines spermine^4+^ and spermidine^3+^ that are abundantly found in living organisms and aid in the packaging of cellular DNA by lowering the free energy of transition required for condensation (21). Within the polyamine class, it was demonstrated that DNA collapse occurs once ∼90% of phosphate charge neutralization has occurred (22) and that polyamine structure, allied with charge, plays a role within condensation transitions (21). Polyamines of varying aliphatic linker length have recently been incorporated into substitution inert tri-platinum(II) complexes (Triplatin) and these highly cationic agents are capable of aggregating nucleic acids (both RNA and DNA) through clamping interactions along the phosphate backbone and across the minor groove of DNA (23–25). Evidence of condensation by Triplatin agents was identified by turbidity and atomic force microscopy (AFM) analysis with DNA (24), particularly poly[d(A-T)_2_] (23), undergoing a B ⟶ ψ transition during the collapse. Another substitutionally inert complex cation, cobalt(III) hexammine [Co(NH_3_)_6_]^3+^, has undergone extensive study for its ionic surface-binding, condensation and dehydration properties with DNA (26,27). Although [Co(NH_3_)_6_]^3+^ can also induce a B ⟶ ψ transition in short oligonucleotide sequences, aggregation of long DNA polymers occurs without modification to the secondary B-DNA conformation (28). Furthermore, while the morphology of DNA condensates with [Co(NH_3_)_6_]^3+^ are commonly toroidal in structure, non-toroidal (rod-like) structures are observed in the presence of alcohol solvents. A further example of metal complex-promoted DNA condensation was recently identified in *di*-Fe^2+^ supramolecular helicates; these 4+ cationic agents bind DNA non-covalently and are capable of inhibiting DNA-processing enzymes including RNA polymerase, DNA topoisomerase I, deoxyribonuclease I, along with a variety of restriction endonucleases (29). Polycations can also induce alternative changes in B-DNA configuration; a reversible B ⟶ A transition, regulated by dehydration, was recently evidenced in short G-C rich oligonucleotide sequences exposed to a poly(allylamine)-*graft*-dextran synthetic cationic polymer (16). Finally, although exceptions to the ≥3+ cationic valence requirement for DNA collapse are rare, both Mn^2+^ and the putrescine^2+^ cations are known to aggregate DNA but only at high (millimolar) concentration (30).

In this study we report the synthesis of a new class of DNA binding scaffold incorporating opioid derivatives. Opioids are naturally derived alkaloids from the opium poppy, *Papaver somniferum*, and serve as one of the major drug classes within pharmaceutical chemistry widely known for their potent analgesic properties (31). Morphine was the first opioid isolated from the poppy and is the most abundant opioid interacting with three principal opioid receptors located in the central nervous system (CNS): mu (μ, MOR), kappa (κ, KOR) and delta (δ, DOR). The interaction of opioids with CNS receptors has led toward the development of synthetic analogues (including heterocodeine, buprenorphine and naltrexone) and to more heavily modified structures containing multiple opiate substituents. Morphine congeners can also be extracted from the poppy (e.g. codeine, thebaine and oripavane) and offer a range of bio-renewable derivatives to support proposed SAR studies. Our group has recently developed a high-throughput ethidium bromide (EtBr) displacement screen to identify potential new DNA binding molecules (32) and, as such, identified a synthetic *C*_3_-symmetric morphine molecule (**MC3**), containing a mesitylene bridging ligand, as a lead compound that was subsequently found to induce DNA condensation at low micromolar concentration. Our motivation for developing heterocodeine (**HC3**) and oripavine (**OC3**) analogues stemmed from structural analysis of **MC3** that focused on the 6′-OH group in the morphine C ring as a possible hydrogen(H)-bonding site for phosphate coordination at the nucleic acid backbone. If this were the case, masking this H-bond through the introduction of methyl groups in the form of methoxy substituents present in heterocodeine (**H**) and oripavine (**O**) should render the DNA binding properties negligible. In addition, as oripavine is a natural product, and commercially available, a short and direct synthesis of the *C*_3_-symmetry target was envisaged. Surprisingly, however, enhanced EtBr displacement was observed for both **HC3** and **OC3** agents, thus implying only a limited role for 6′-OH phosphate interaction in the binding mechanism. Consequently, the DNA binding mode and condensation properties of this class of molecule were elucidated using an array of biophysical techniques including turbidity, viscosity, thermal melting, circular dichroism (CD) spectroscopy, electrophoresis and AFM analysis and are reported herein.

## MATERIALS AND METHODS

### Materials synthesis and characterization

Chemicals and reagents were sourced from Sigma-Aldrich and were used without any further purification required. High pressure (or high performance) liquid chromatography (HPLC) grade chloroform, methanol and acetonitrile were used with no further purification. All other solvents were used as supplied. Morphine and oripavine were provided from Johnson Matthey MacFarlan Smith Ltd. Thin layer chromatography was performed on Fluka Silica gel (60 F254) coated on aluminium plates. The TLC plates were visualized using UV light. Davisil 60 Å silica gel was used for column chromatography. ^1^H and ^13^C NMR spectra were obtained on a Bruker AC 400 and 600 MHz NMR spectrometer. The pH was monitored by a Mettler Toledo InLab Expert Pro-ISM pH probe. Electrospray ionization mass spectra (ESI-MS) were recorded using a Thermo Fisher Exactive Orbitrap mass spectrometer coupled to an Advion TriVersa Nanomate injection system with samples being prepared in 100% HPLC-grade acetonitrile prior to ESI-MS analysis. CD spectrometry was conducted on an Applied Photophysics Chirascan plus qCD spectrometer with samples being prepared in acetonitrile.

#### Morphine-C_3_ (MC3)

Morphine (0.573 g, 2.01 mmol) and potassium carbonate (1.110 g, 8.03 mmol) were suspended in acetonitrile (ACN) (30 ml) and heated to reflux. 2,4,6-*Tris-*(bromomethyl)-mesitylene (0.268 g, 0.66 mmol) was added in small aliquots to the reaction mixture and vigorously stirred overnight (18 h) under reflux conditions. After allowing the reaction mixture to cool to room temperature (RT), the reaction solvent was removed by rotary evaporation and crude product dissolved in dichloromethane (DCM) (50 ml). The organic layer was washed with d.H_2_O (40 ml) and the aqueous layer extracted with DCM (3 × 20 ml). All organic layers were combined, then washed with d.H_2_O (3 × 20 ml) and with a saturated brine solution (20 ml). The organic layer was dried over magnesium sulphate, filtered and solvents removed by rotary evaporation. The crude product was purified by column chromatography (SiO_2_, 95:1:1 to 92:8:1 CH_2_Cl_2_:MeOH:NH_4_OH). The title compound **MC3** was isolated as a white solid in 50% yield (0.338 g, 0.33 mmol). mp. 187–189°C. ^1^H NMR (600 MHz, CDCl_3_) δ: 6.80 (d, *J* = 8.2 Hz, 3H); 6.58 (d, *J* = 8.2 Hz, 3H); 5.69 (ddt, *J* = 9.9, 3.2, 1.4 Hz, 3H); 5.30–5.27 (m, 3H); 5.21 (d, *J* = 10.5 Hz, 3H); 5.05 (d, *J* = 10.4 Hz, 3H); 4.85 (dd, *J* = 6.5, 1.1 Hz, 3H); 4.17–4.13 (m, 3H); 3.35 (s, 3H); 3.05 (d, *J* = 18.7 Hz, 3H); 2.66 (s, 3H); 2.61–2.56 (m, 3H); 2.48 (s, 9H); 2.44 (s, 9H); 2.41 – 2.37 (m, 3H); 2.31 (dd, *J* = 18.7, 6.2 Hz, 3H); 2.05 (td, *J* = 12.4, 5.0 Hz, 3H); 1.88 (d, *J* = 11.3 Hz, 3H). ^13^C NMR (151 MHz, CDCl_3_) δ: 147.59, 141.43, 139.43, 133.41, 131.82, 131.44, 128.19, 119.61, 116.36, 91.27, 77.22, 77.00, 76.79, 67.32, 66.46, 58.93, 46.47, 43.09, 42.96, 40.81, 35.78, 20.64, 15.98. IR (ATR, cm^−1^): 2907, 1632, 1602, 1492, 1443, 1247, 1200, 1157, 1118, 1099, 1034, 983, 940, 833, 784, 766, 731. ESI-MS: [**MC3**] 1012 m/z. [α]_D_ = −85° (c = 0.154, CHCl_3_, 589 nm, 25°C).

#### Oripavine-C_3_ (OC3)

A flask was charged with oripavine (0.595 g, 2.00 mmol), tetrabutylammonium hydroxide (40% aqueous solution, 18 ml) and DCM (6 ml) and stirred under nitrogen for 30 min. A solution of 2,4,6-*tris-*(bromomethyl)-mesitylene (0.269 g, 0.66 mmol) in DCM (4 ml) was added and the biphasic reaction mixture was stirred for 6 h at RT. The reaction solution was transferred into d.H_2_O (150 ml) and washed with DCM (4 × 10 ml). Organic layers were combined and washed with aqueous NaOH solution (0.1 M, 2 × 20 ml) followed by d.H_2_O (3 × 20 ml) then saturated brine solution (20 ml). The organic layer was dried over magnesium sulphate, filtered and solvents removed by rotary evaporation. The crude product was purified by column chromatography (SiO_2_, 95:1:1 to 92:8:1 CH_2_Cl_2_:MeOH:NH_4_OH). The title compound **OC3** was isolated as a golden yellow solid in 47% yield (0.329 g, 0.31 mmol). mp 174–176°C. ^1^H NMR (600 MHz, CDCl_3_) δ: 6.73 (d, *J* = 8.1 Hz, 3H); 6.58 (d, *J* = 8.1 Hz, 3H); 5.56 (d, *J* = 6.4 Hz, 3H); 5.28 (s, 3H); 5.25 (d, *J* = 10.7 Hz, 3H); 5.17 (d, *J* = 10.7 Hz, 3H); 5.03 (d, *J* = 6.4 Hz, 3H); 3.62 (d, *J* = 6.6 Hz, 3H); 3.59 (s, 9H); 3.32 (d, *J* = 18.0 Hz, 3H); 2.83 (td, *J* = 12.7, 3.3 Hz, 3H); 2.68 (dd, *J* = 18.1, 7.0 Hz, 3H); 2.63 (dd, *J* = 12.7, 4.6 Hz, 3H); 2.47 (s, 9H); 2.46 (s, 9H); 2.20 (td, *J* = 12.6, 5.1 Hz, 3H); 1.78–1.75 (m, 3H). ^13^C NMR (151 MHz, CDCl_3_) δ: 152.96, 146.07, 142.13, 139.65, 133.99, 132.59, 132.06, 128.73, 119.46, 117.48, 111.73, 96.09, 89.16, 89.11, 77.37, 77.16, 76.95, 67.74, 61.07, 55.04, 46.25, 46.16, 42.57, 37.11, 29.93, 16.02. IR (ATR, cm^−1^): 2908, 1605, 1491, 1437, 1368, 1331, 1302, 1231, 1143, 1105, 1066, 1021, 987, 914, 867, 812, 767, 748, 698. ESI-MS: [**OC3**]^+^ 1048 m/z. [α]_D_ = −88° (c = 0.12, CHCl_3_, 589 nm, 25°C).

#### Heterocodeine (33,34)

Reaction carried out on parallel synthesizer. Potassium hydride (4.421 g, 110.23 mmol) was prepared in the reaction vessel under nitrogen flux and washed with dry hexane, suspended in dry tetrahydrofuran (THF) (150 ml) over ice. A solution of morphine (2.862 g, 10.03 mmol) in THF (30 ml) was added slowly over 30 min to the reaction under a nitrogen atmosphere and the resulting solution was allowed to stir at RT for 16 h. Methyl iodide (1.710 g, 0.75 ml, 12.05 mmol) was added to the reaction slowly over 15 min and reaction left stirring for 4 h. The reaction was quenched slowly with a mixture of THF/H_2_O (10:1) at 0°C. The solution was neutralized to pH 7.0 with 2 M HCl and volatiles were then removed by rotary evaporation. The pH was adjusted to 8.0 by the addition of 1M NaOH and the aqueous layer extracted with chloroform/isopropanol (3:1, 3×25 ml). The resulting organic layer was washed with H_2_O (4×30 ml) and a final wash with saturated brine solution (20 ml). The organic layer was dried over magnesium sulphate, filtered and solvents removed by rotary evaporation. The crude product was purified by column chromatography (SiO_2_, 95:1:1 to 92:8:1 CH_2_Cl_2_:MeOH:NH_4_OH), heterocodeine was isolated as a white solid in 25% yield (756 mg, 2.53 mmol). ^1^H NMR (600 MHz, CDCl_3_) δ: 6.57 (d, *J* = 8.1 Hz, 1H); 6.41 (d, *J* = 8.1 Hz, 1H); 5.64 (ddt, *J* = 9.9, 3.2, 1.5 Hz, 1H); 5.26 (dt, *J* = 9.8, 2.7 Hz, 2H); 4.91 (dd, *J* = 5.8, 1.3 Hz, 1H); 3.72 (td, *J* = 5.5, 2.3 Hz, 1H); 3.45 (s, 3H); 3.32 (dd, *J* = 6.3, 3.2 Hz, 1H); 2.97 (d, *J* = 18.6 Hz, 1H); 2.63 – 2.49 (m, 2H); 2.43 – 2.31 (m, 4H); 2.23 (dd, *J* = 18.7, 6.4 Hz, 1H); 1.99 (td, *J* = 12.4, 5.1 Hz, 1H); 1.88–1.79 (m, 2H).

#### Heterocodeine-C_3_ (HC3)

A flask was charged with heterocodeine (0.700 g, 2.34 mmol), tetrabutylammonium hydroxide (40% aqueous solution, 20 ml) and DCM (8 ml) and stirred under nitrogen for 30 min. A solution of 2,4,6-*tris-*(bromomethyl)-mesitylene (0.269 g, 0.66 mmol) in DCM (4 ml) was added and the mixture was stirred for 6 h at RT. The reaction solution was transferred into d.H_2_O (150 ml) and washed with DCM (4 × 10 ml). Organic layers were combined and washed with aqueous NaOH solution (0.1 M, 2 × 20 ml) followed by d.H_2_O (3 × 20 ml) then a saturated brine solution (20 ml). The organic layer was dried over magnesium sulphate, filtered and solvents removed by rotary evaporation. The crude product was purified by column chromatography (SiO_2_, 95:1:1 to 92:8:1 CH_2_Cl_2_:MeOH:NH_4_OH), **HC3** was isolated by column chromatography as a white solid in 13% yield (95 mg 0.09 mmol). mp. 164–166°C. ^1^H NMR (400 MHz, CDCl_3_) δ: 6.73 (d, *J* = 8.1 Hz, 3H); 6.49 (d, *J* = 8.1 Hz, 3H); 5.71 (d, *J* = 9.9 Hz, 3H); 5.32 (dt, *J* = 10.0, 2.7 Hz, 3H); 5.27–5.16 (m, 6H); 5.00 (d, *J* = 5.1 Hz, 3H); 3.80 (dd, *J* = 5.4, 2.7 Hz, 3H); 3.51 (s, 9H); 3.36 (dd, *J* = 5.9, 3.1 Hz, 3H); 3.04 (d, *J* = 18.7 Hz, 3H); 2.69–2.65 (m, 3H); 2.61–2.56 (m, 3H); 2.52 (s, 9H); 2.44 (s, 9H); 2.40 (d, *J* = 3.4 Hz, 3H); 2.31 (dd, *J* = 18.7, 6.3 Hz, 3H); 2.04 (td, *J* = 12.4, 5.0 Hz, 3H); 1.93 (d, *J* = 11.0 Hz, 3H).^13^C NMR (101 MHz, CDCl_3_) δ: 148.88, 141.35, 139.66, 132.09, 131.46, 131.01, 128.56, 128.18, 119.02, 118.34, 89.14, 77.48, 77.36, 77.16, 76.84, 75.67, 68.01, 59.06, 56.92, 53.58, 46.68, 43.61, 43.26, 43.19, 41.23, 36.06, 29.82, 20.66, 16.10. IR (ATR, cm^−1^): 2905, 2798, 1632, 1601, 1492, 1442, 1247, 1199, 1104, 984, 941, 831, 787, 768, 727, 679. [α]_D_ = −185° (c = 0.08, CHCl_3_, 589 nm, 25°C).

### DNA binding experiments

#### Competitive ethidium bromide displacement assay

The DNA binding affinity of the tripodal series was determined over a 5 h time period using calf-thymus DNA (ctDNA, Ultra-Pure Invitrogen, 15633019) and synthetic alternating co-polymers poly[d(A-T)_2_)] (Sigma Aldrich, P0883) and poly[d(G-C)_2_] (Sigma Aldrich, P9389) by ethidium bromide fluorescence quenching in a similar manner to the high-throughput method previously reported by Kellett *et al*. (35). Each drug concentration was measured in triplicate, on at least two separate occasions, and the apparent binding constants were calculated using *K*_app_ = *K*_b_ × 12.6/C_50_ where *K*_b_ = 8.80 × 10^6^ M(bp)^−1^ (*K*_app_ = apparent binding constant).

#### Viscosity studies

Experiments were conducted in a similar manner to the method reported previously using DV-II-Programmable Digital Viscometer equipped with Enhanced Brookfield UL Adapter at room temperature by gradually increasing the [compound/DNA] ratios from 0.02–0.20 (36).

#### Thermal melting studies

The thermal melting of DNA of varying %AT content was conducted using an Agilent Cary 100 dual beam spectrophotometer equipped with a 6 × 6 Peltier multicell system with temperature controller. The protocol was previously developed within the Kellett group and reported elsewhere (6,7).

### DNA condensation studies (dsDNA)

#### Turbidity investigation (ctDNA)

This method was adapted from the Brabec laboratory (24). Absorbance was initially measured at 260 nm in order to give a final measurement of ∼0.5 units and the concentration of ctDNA was determined using the extinction coefficient ϵ_260_ = 12 824 M(bp)^−1^ cm^−1^. The turbidity of the ctDNA solution was determined spectrophotometrically by monitoring the absorbance of DNA at both 350 nm and 260 nm at 25°C using an Agilent Cary 100 dual beam spectrophotometer equipped with a 6 × 6 Peltier multicell system with temperature controller and stirring mechanism. In a final volume of 3 ml containing ∼40 μM ctDNA, 1 mM PBS buffer (pH 7.0) and 25 mM NaCl, varying concentrations of **MC3, OC3** and **HC3** (2.5, 5, 7.5, 10, 12.5, 15, 20, 25, 30, 35, 45, 50, 65, 75, 80, 90, 100 μM) were titrated into quartz cuvettes. Between each aliquot, solutions were mixed thoroughly and allowed to incubate at 25°C until the absorbance equilibrated and values obtained remained constant.

#### DNA condensation investigation (pUC19 scDNA)

The ability of the organic compounds to condense supercoiled plasmid DNA was determined using a method previously published by this laboratory with minor changes being made (23). Tripodal opiates were initially prepared in DMF and further diluted in 80 mM HEPES buffer (Fisher). Reactions were carried out according to the following general procedure: in a total volume of 20 μl using 80 mM HEPES buffer (pH 7.2) with 25 mM NaCl, 400 ng pUC19 (NEB, N3041) and varying concentrations of test compound (5, 10, 20 and 30 μM), samples were incubated at 37°C for both 5 and 12 h. Reactions were quenched by adding 6x loading buffer (Fermentas) containing 10 mM Tris-HCl, 0.03% bromophenol blue, 0.03% xylene cyanole FF, 60% glycerol, 60 mM EDTA and samples were loaded onto an agarose gel (1.2%) containing 3 μl EtBr. Electrophoresis was completed at 60 V for 1 h in 1x TAE buffer.

#### DNA condensation investigation in the presence of non-covalently bound recognition elements (pUC19 scDNA)

This protocol was carried out as previously reported by this group with minor changes made (37). Briefly, 400 ng pUC19 was incubated with 25 mM NaCl, and 20 μM of either methyl green, netropsin or hexamine cobalt(III) chloride in 80 mM HEPES buffer (pH 7.2) for 30 min at 37°C. Sample tubes were then vortexed and varying concentrations of test compound were added (5,10, 20 and 30 μM). The reaction mixture was further incubated at 37°C for 5 h. The reaction was then quenched and subjected to gel electrophoresis as previously described.

#### Investigation of DNase I inhibition by DNA condensation (pUC19 scDNA)

pUC19 DNA (400 ng) was initially exposed to 75, 100, 200 and 300 μM of test compounds with 25 mM NaCl in a total volume of 20 μl using 80 mM HEPES buffer (pH 7.2) for 5 h at 37°C. The condensed DNA was then treated with the endonuclease, DNase I (NEB, M0303S), for 10 min at 37°C and heat inactivated at 75°C for 10 min. Samples were then loaded onto an agarose gel (1.5%) containing 3 μl EtBr and electrophoresis was completed at 50 V for 40 min in 1x TAE buffer.

#### CD spectroscopy

Opioid profiles (**MC3** and **OC3**) and opioid–DNA interactions were analysed using Starna quartz cuvettes in 10 mM PBS solution (pH 7.0) in the presence of 25 mM NaCl. Salmon testes DNA (stDNA, Sigma Aldrich, D1626) was initially quantified using the extinction co-efficient ϵ_260_ = 12 824 M(bp)^−1^ cm^−1^ to give a working solution with final stDNA concentration of ∼100 μM. The investigation was conducted in the range of 200–400 nm and measurements were recorded at a rate of 1 nm per second. A total of 100 μM stDNA solution was incubated with **MC3, OC3** and control agents hexammine cobalt(III) chloride and spermine at *r* = 0.1 (*r* being the ratio [drug]/[DNA]) over a 7 h period at 37°C. The DNA free CD spectra for **MC3** and **OC3** are shown in Supplementary Figure 13.

### DNA condensation studies (linear dsDNA)

#### PCR primer design

Primers were designed (Eurofins Genomics) such that it was possible to generate a 742 bp long sequence of linear dsDNA from the pUC19 vector encompassing the lacZα gene. The short nucleotide sequence was generated through PCR (35 cycles) with 1 ng pUC19 plasmid using 2x MyTaq Red Mix (Bioline) at an annealing temperature of 66°C and the band generated was compared to a 50 bp DNA ladder (Invitrogen, 10416014).

Forward: 5′-TCGCGCGTTTCGGTGATGACGG-3′

Reverse: 5′-CCGCTCGCCGCAGCCGAACG-3′

#### DNA condensation investigation

A total of 400 ng of the 742 bp transcript was then treated under identical conditions as described (5 h incubation at 5, 10, 20 and 30 μM) and samples were loaded onto an agarose gel (1.2%) containing EtBr. Electrophoresis was completed at 70 V for 40 min in 1x TAE buffer.

### Endonuclease enzyme Inhibition

#### Endonuclease optimization on duplex DNA

A total of 400 ng of linear dsDNA was treated with 1 μl of EcoRI (NEB, R0101S), BamHI (NEB, R0136S), SalI (NEB, R0138S) and HindIII (NEB, R0104S), respectively, at 37°C overnight to verify the presence of their recognition sites within the short 742 bp sequence. All enzymes induced double stranded nicks to this sequence as evidenced by agarose gel electrophoresis.

#### Microfluidic analysis of endonuclease inhibition using an Agilent Bioanalyzer DNA 1000 chip

This assay was conducted as previously described by the Kellett Group with small changes being made (23). A total of 400 ng of the PCR fragment was pre-treated with 10, 25, 50, 100 and 200 μM of either **MC3, HC3** or **OC3**. Subsequent digestion experiments were performed by incubating drug treated and un-treated DNA with 1 μl of EcoRI, BamHI, SalI and HindIII overnight and heat deactivated as per NEB guidelines. The reactions of EcoRI, BamHI, SalI and HindIII, in the presence and absence of tripodal scaffolds, were then examined using the Agilent DNA 1000 microfluidic chip (Agilent 5067-1504) with data being collected on the Agilent Bioanalyzer 2100 platform.

### Atomic force microscopy (AFM)

AFM was used to determine the morphology of the DNA condensates induced by a morphine *C*_3_ opiate to support the effects of condensation at low concentrations. AFM samples were prepared according to the following general procedure: in a total volume of 10 μl final concentrations of 3 ng/μl of pUC19 (NEB, N3041) or linearized pUC19, 5 mM MgCl and varying concentrations of test compound, were incubated at 37°C for 1 h. Ten microliters of each sample was pipetted directly on to freshly cleaved mica and allowed to incubate for 5 min followed by rinsing with 500 μl of water. The samples were dried under compressed air for a period of 1 h. AFM examinations were performed in ambient air with a commercial microscope (Dimension 3100 controlled by a Nanoscope IIIa controller, Digital Instruments), in tapping-mode, using standard unmodified silicon cantilevers (BudgetSensors, Windsor Scientific Ltd.) with a 40 N/m force constant. Topographic images are recorded at a scanning rate of 1–2 Hz, and a resonance frequency of about 300 kHz (nominal value). Images were processed using the WSxM software (38) to remove the background slope and normalize the z-scale across all images, no additional filtering was performed.

### Influence of pH and ionic strength on condensation

#### Influence of pH on condensation

Absorption measurements spectra were initially measured at 260 nm to give a final absorbance of ∼0.4 units and the concentration of ctDNA was determined using the extinction co-efficient ϵ_260_ = 12 824 M(bp)^−1^ cm^−1^. The condensation aggregates of the ctDNA solution was determined spectrophotometrically by monitoring the absorbance of DNA at both 350 nm and 260 nm at 25°C using an Agilent Cary 100 dual beam spectrophotometer equipped with a 6 × 6 Peltier multicell system with temperature controller. In a final volume of 5 ml containing ∼30 μM ctDNA, nuclease free water (pH 7) and 25 mM NaCl, containing 35 μM **OC3**, the pH of solution was adjusted with 1 M HCl and 1 M NaOH to basic and acidic conditions as required using a Mettler Toledo Inlab expert Pro-ISM pH probe. One hundred microliters aliquots were titrated into quartz cuvettes and between each pH adjustment solutions were mixed thoroughly and allowed to incubate at 25°C until the absorbance equilibrated and values obtained remained constant.

#### DNA condensation in acidic and basic buffers

A total of 400 ng of pUC19 DNA was treated as previously stated with slight modifications (5 h incubation at 5, 10, 20, 30 and 50 μM). Samples were incubated in acidic and basic buffers at pH 4.0 and 8.0, respectively. Sodium acetate (NaOAc) and Tris buffers were prepared and adjusted with HCl and NaOH accordingly to achieve the desired pH. Samples were loaded onto an agarose gel (1.2%) containing 3 μl EtBr. Electrophoresis was completed at 70 V for 40 min in 1x TAE buffer.

## RESULTS AND DISCUSSION

### Synthesis of opioid scaffolds

**Figure 1.**
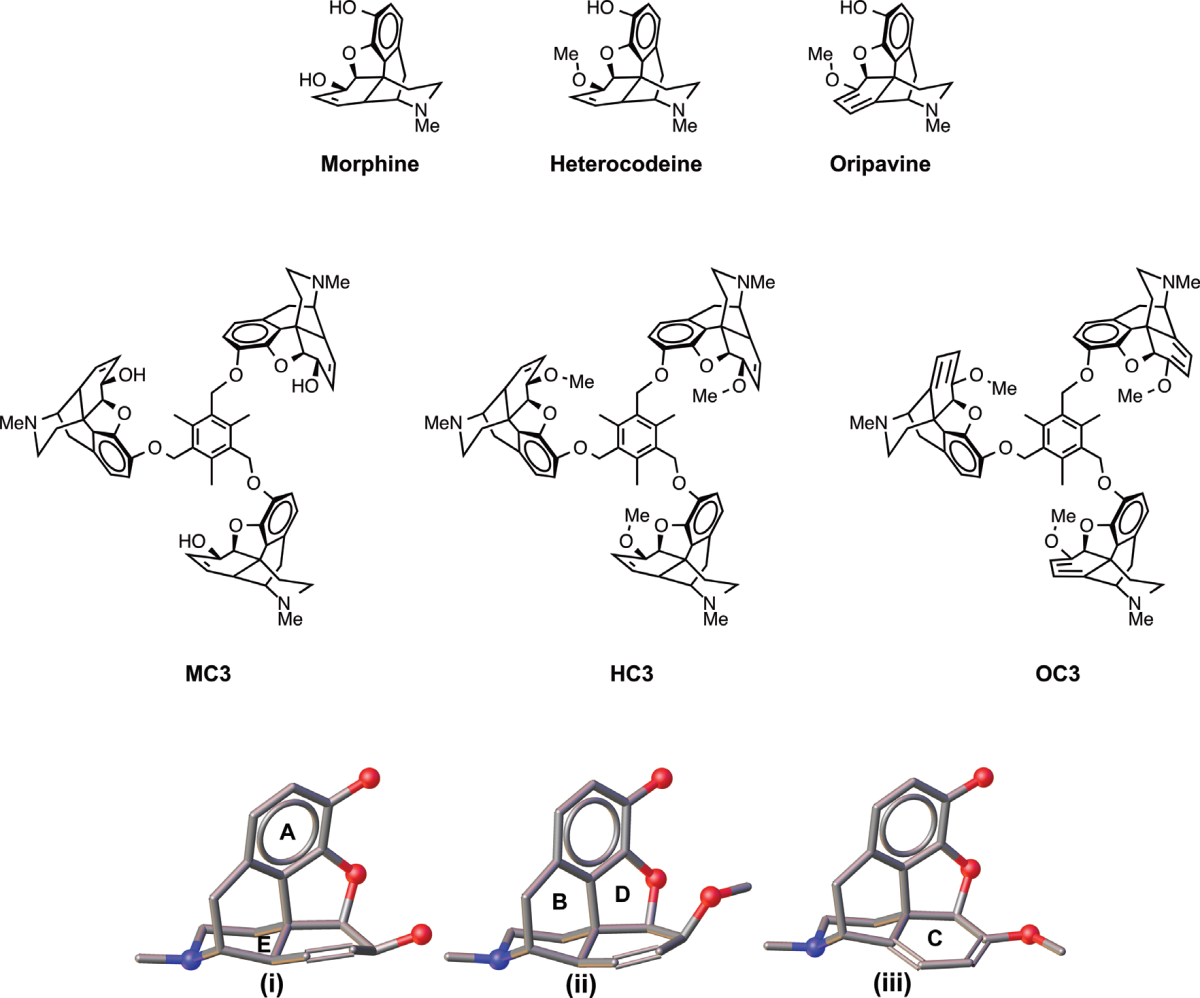
Molecular structures of morphine, heterocodeine and oripavine along with *C*_3_-symmetic opioid molecules developed in this study: morphine (**MC3**), heterocodeine (**HC3**) and oripavine (**OC3**). Left to right, morphine, heterocodeine and oripavine scaffolds. Structures **(i), (ii)** and **(iii)** show the geometries of each scaffold (morphine, heterocodeine and oripavine respectively), with labeled ring substituents **A–E**, modified from X-ray structures reported in the CSD.

Morphine scaffolds **MC3, HC3** and **OC3** were generated and their molecular structures are shown in Figure 1. Treating 1 equivalent of 2,4,6-*tris*-(bromomethyl)-mesitylene with 3 equivalents of either morphine (**M**), heterocodeine (**H**) or oripavine (**O**) yielded the respective *C*_3_-symmetric opioid compounds **MC3, HC3** and **OC3**. These were isolated as yellow or white solids after purification by column chromatography. A satisfactory yield for both **MC3** (50%) and **OC3** (48%) was obtained, however, **HC3** could only be isolated in a low yield (13%). Indeed, generation of the **HC3** opioid was restricted due to the poor synthetic conversion of morphine to heterocodeine with yields <25% (39) that leads to a low overall yield for **HC3** from morphine. *C*_1_ and *C*_2_ opiate analogues (**MC1, MC2, OC1** and **OC2**) were synthesized in a similar manner to the *C*_3_ congeners using α-2-chloroisodurene (**MC1** and **OC1**) and 2, 4-*bis*-(chloromethyl)-1,3,5-trimethylbenzene (**MC2** and **OC2**), respectively, of each opioid derivative (Supplementary Section S-7). The series of compounds for this study were all prepared in either a one- or two-step synthesis that enabled rapid access to samples for evaluation in biological investigations. As part of this work, a green chemistry metric analysis (40) has been performed (Supplementary Section S-10) with the aim of identifying aspects of the synthesis that can be improved in future studies (41,42). This will ensure that further efforts to improve the yield for the above reactions can be in tandem with the development of greener methodologies. All compounds were characterized by ^1^H and ^13^C nuclear magnetic resonance (NMR, Supplementary Section S-1) and attenuated total reflectance (ATR) Fourier transform infrared (FTIR) spectroscopies, and by ESI-MS. Solution-based NMR studies in deuterated chloroform were performed to elucidate whether all three opiate units were on the same face of the molecule (*C*_3_) or whether rotation of one opiate ‘arm’ was rapidly occurring at room temperature. To test the latter, a temperature study was conducted. This confirmed a *C*_3_-symmetric opioid compound was formed for each of the *tris* target compounds; a single set of peaks in the ^1^H NMR was observed for the opioid substructure for **MC3, HC3** and **OC3** in both 1D and 2D NMR studies. As the probe temperature was decreased from 293 K to 243 K, no changes in the proton NMR were detected, supporting the *C*_3_ symmetry of the compounds as a stable configuration.

### Condensation of duplex DNA

**Figure 2.**
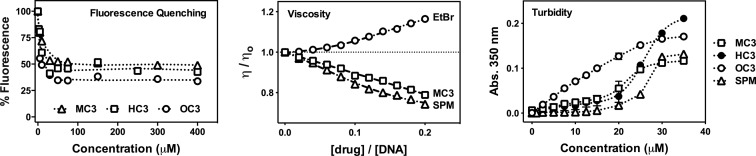
Competitive fluorescence quenching of ethidium bromide bound to calf thymus (ct-DNA) by opioid drugs **MC3, HC3** and **OC3**, viscosity properties of **MC3**, ethidium bromide and spermine (**SPM**) exposed to salmon testes dsDNA, and turbidity profiles of CT-DNA in the presence of titrated *C*_3_ opioids and spermine. Data points being displayed as an average of triplicate measurement for fluorescence quenching and turbidity measurements.

*C*_3_ opiate binding interactions with duplex DNA were first identified using a saturated EtBr fluorescence quenching study with calf thymus DNA (ct-DNA) (Figure 2). In our initial high-throughput screen, which involved a series of novel opioid scaffolds, **MC3** was identified as a potentially new DNA-binding molecule. Our motivation for developing **HC3** and **OC3** isomers stemmed from structural analysis on **MC3** that focused on the 6′-OH group in the morphine C ring as a possible H-bonding site for phosphate coordination. Since this group is masked by methylation in heterocodeine (**H**) and oripavine (**O**), we anticipated **HC3** and **OC3** analogues would have negligible DNA binding properties. Unexpectedly, however, enhanced EtBr displacement was observed (Table 1), particularly on poly[d(G-C)_2_] and poly[d(A-T)_2_] co-polymers with apparent binding constants (*K*_app_) of ∼10^6^ M(bp^−1^) and ∼10^7^ M(bp^−1^), respectively.

**Table 1.** Apparent binding constants of opioid *C*_3_ compounds to dsDNA polymers. (N.D. = not detected)

	*K*_app_ M(bp^−1^)			ΔT_M_ °C (± S.D.)	
	Poly A-T (100% A-T)	ct-DNA (58% A-T)	Poly G-C (0% A-T)	Poly A-T (100% A-T)	Poly G-C (0% A-T)
Actinomycin D (35)	N.D.	2.92 × 10^7^	5.35 × 10^7^	−0.32 ± 0.29	12.10 ± 0.95
Netropsin (35)	5.75 × 10^7^	2.5 × 10^6^	N.D.	12.32 ± 0.79	2.83 ± 0.38
MC3	3.03 × 10^5^	6.40 × 10^6^	2.69 × 10^5^	0.38 ± 0.71	2.63 ± 0.58
OC3	3.63 × 10^6^	3.82 × 10^6^	3.5 × 10^7^	−0.17 ± 0.25	0.36 ± 0.17
HC3	9.1 × 10^6^	7.84 × 10^6^	1.15 × 10^7^	0.73 ± 0.18	1.72 ± 0.61

To identify the significance of the *C*_3_ opioid scaffold symmetry toward duplex DNA binding, **OC1, OC2, MC1** and **MC2** compounds were generated and tested using gel electrophoresis experiments (24,35,37). Condensation results, however, revealed negligible *C*_1_ and *C*_2_ activity (Supplementary Figure 14 and 15) confirming the requirement of a tertiary opioid substituent to facilitate efficient nucleic acid coordination at low micromolar concentration. Thermal melting analysis of duplex polymers were then completed (Table 1), and, unlike classic DNA binding agents netropsin and actinomycin D (35), examined previously under identical conditions by this group, no significant stabilization energies were identified for the *tris*-opiate compounds.

In order to probe the nature of DNA binding, viscosity studies with salmon testes DNA (st-DNA) at varying drug load were evaluated (Figure 2). Classical condensation behavior by *C*_3_ opioids was observed whereby all three agents exhibited identical, downward curving, hydrodynamic (*η*/*η*_o_) values. As shown in Figure 2, the profile of **MC3** is similar to the well-studied DNA compaction agent spermine, which was run as a control agent alongside the classical DNA intercalator EtBr. Since condensation of dsDNA can be monitored at 350 nm, a wavelength where nucleic acids do not normally absorb unless condensation/aggregation has occurred (24), UV-vis absorption spectrophotometry was employed to further characterize the condensation process. (Although turbidity of dsDNA can also be monitored at 260 nm, electronic absorption of *C*_3_ opioids in this region precluded this measurement.) All three opioids exhibit concentration-dependent aggregation of dsDNA with binding isotherms of **MC3** and **HC3** being sigmoidal in nature and approximately similar in shape to spermine (Figure 2). **OC3**, however, has a markedly different profile that increases almost linearly between 1–25 μM before reaching a plateau beyond this titration point. Overall, and in comparison to spermine, greater aggregation was observed for *C*_3_ opioids tested up to 25 μM, beyond which, **OC3** and **MC3** scaffolds can be described as having the highest condensation effects in the series.

**Figure 3.**
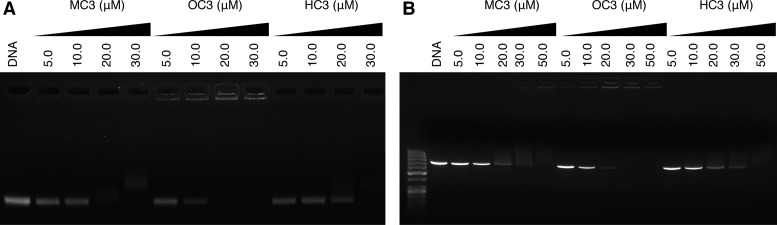
(**A**) Agarose gel electrophoresis of supercoiled (400 ng) and (**B**) a 742 bp dsDNA fragment of pUC19 (400 ng) exposed to increasing concentrations of **MC3, OC3** and **HC3**. Reactions were carried out in the presence of 25 mM NaCl for 5 h at 37°C prior to electrophoretic analysis.

Since condensation by *C*_3_ opioids had been established by viscosity and turbidity measurements, visualization of DNA compaction was then followed by electrophoresis. Samples were titrated against both supercoiled pUC19 plasmid DNA and a 742 bp dsDNA fragment amplified from pUC19 encompassing the lacZα gene, before incubation for 5 h prior to analysis by agarose gel electrophoresis (Figure 3). All three opioid scaffolds were found to condense both supercoiled and linear dsDNA; **MC3** and **HC3** have similar profiles with the onset of aggregation at 20 μM, beyond which native DNA bands become fainter in appearance or disappear entirely from view, thus reflecting total condensation. **OC3**, however, exhibits improved condensation effects in comparison to morphine and heterocodeine scaffolds. Here, the initiation of aggregation can be visualized at 5 μM with the condensation process being completed at 20 μM and 30 μM on supercoiled plasmid and linear DNA, respectively. Indeed, on comparing the gels, it is evident that opioid-induced aggregation of supercoiled pUC19 (Figure 3A) occurs more efficiently than for linear dsDNA (Figure 3B).

**Figure 4.**
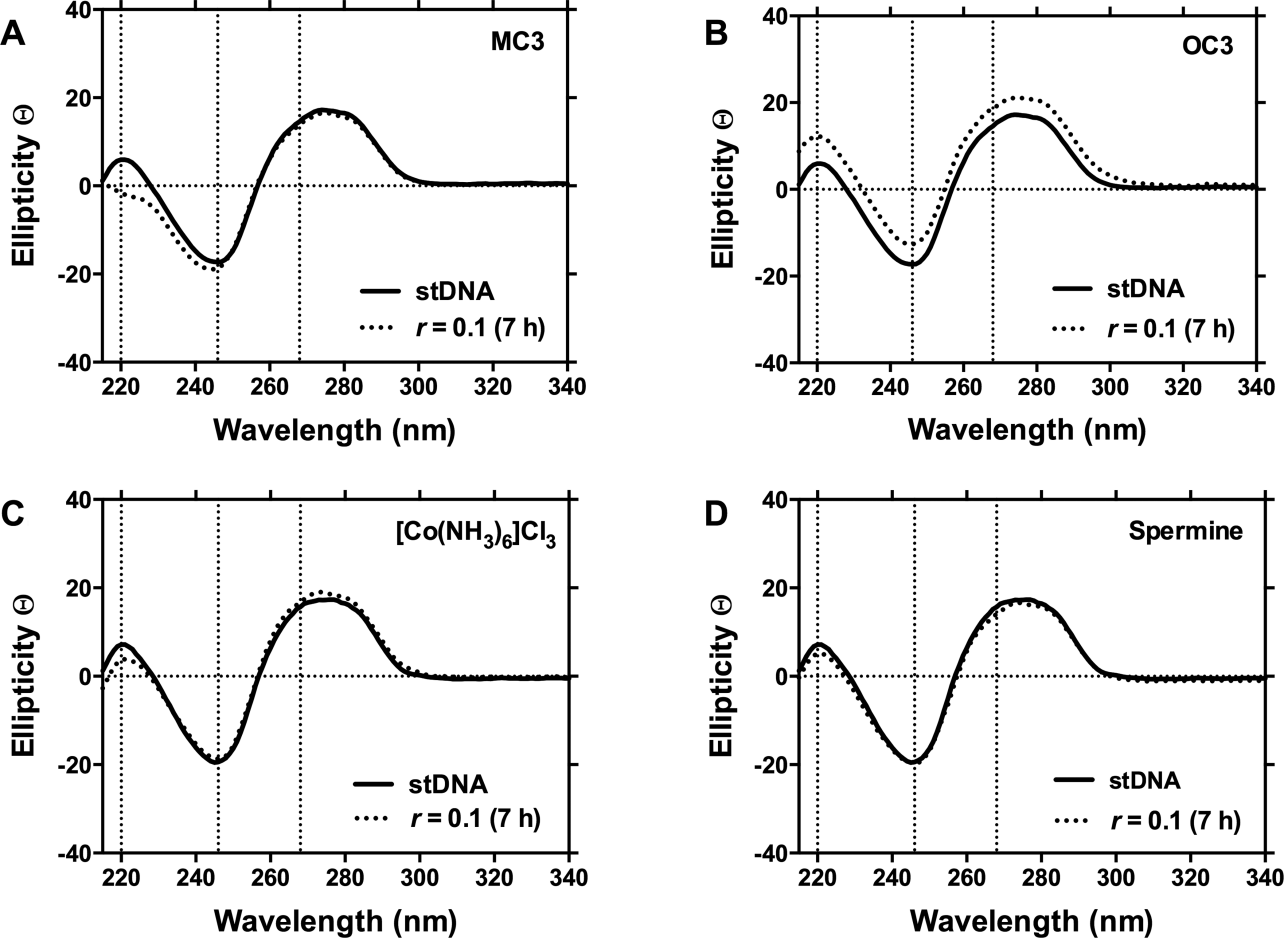
Circular dichroism (CD) spectra of st-DNA treated with **MC3, OC3** and electrostatic DNA binding controls hexammine cobalt(III) chloride and spermine over 7 h at *r* ([opioid] / [DNA]) values of 0.1.

To further probe the condensation mechanism, the interaction of *C*_3_ opioids with pUC19 were examined in the presence of non-covalently bound recognition elements netropsin, which is well-characterized to bind within the minor groove (43), and methyl green – an agent that binds at the major groove (44). In these experiments plasmid DNA was pre-exposed to 20 μM of each recognition element prior to the introduction of opioid sample (5–30 μM). No differences were detected in the condensation process between the control (opioid + pUC19) and tested samples in any case, indicating interactions within the minor and major grooves are unlikely as prospective recognition sites for *C*_3_ opioid binding (data not shown). Indeed, no evidence of minor or major groove binding, or of intercalation could be found in the CD spectra of **MC3** and **OC3** with st-DNA (Figure 4). It was further noted that no conformational change was induced upon opioid binding and condensation. Thus, the profiles for both *C*_3_ scaffolds do not yield spectral shifts expected of classical DNA binding agents but, instead, are similar to surface-binding condensation agents spermine and [Co(NH_3_)_6_]Cl_3_ (45) when tested with long DNA polymers. Finally, while the spectrum of **HC3** could not be examined owning to its limited solubility in CD accessible solvents, it is reasonable to assume the binding interaction should not deviate from that observed from **MC3** or **OC3**.

### Influence of pH and ionic strength on condensation

**Figure 5.**
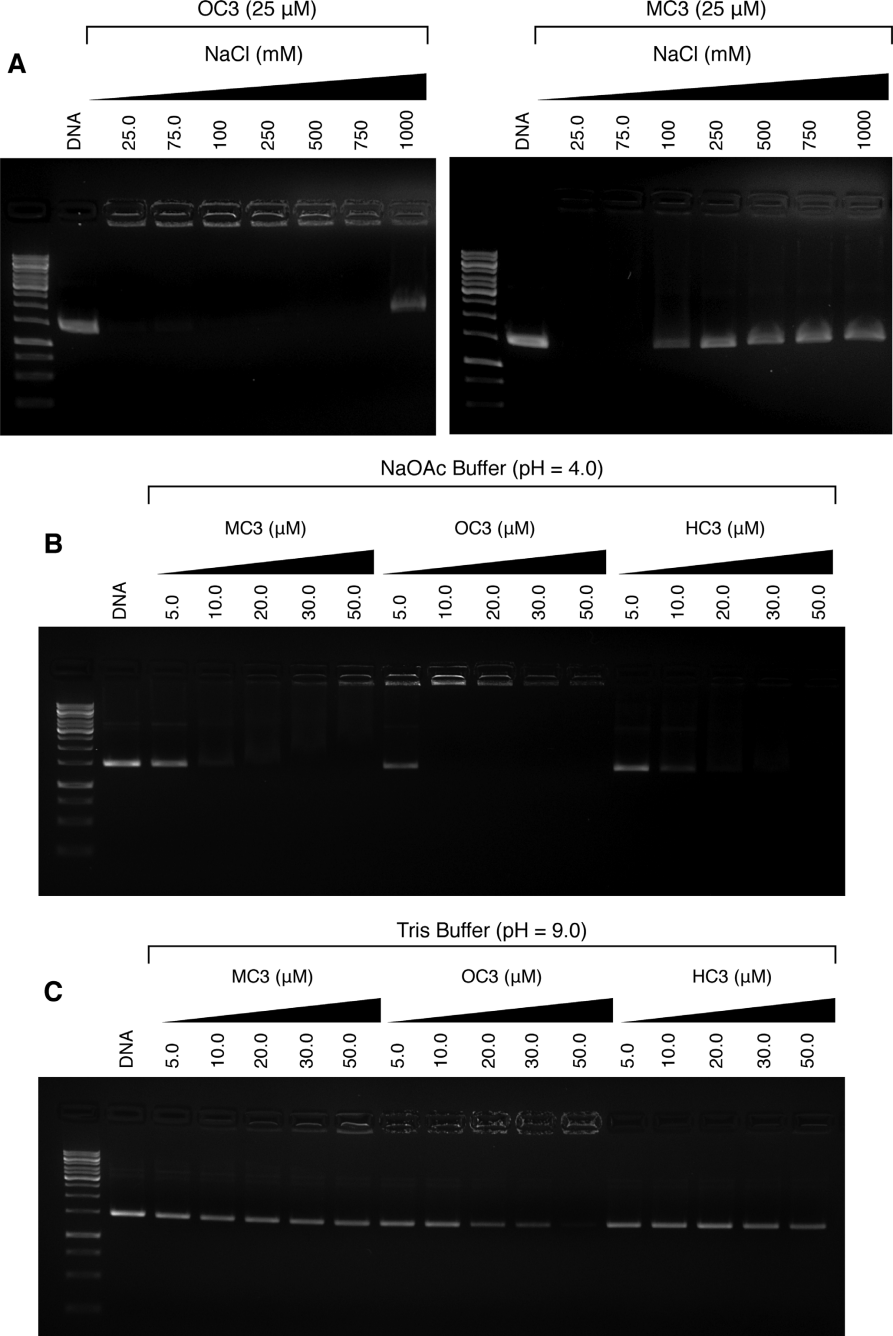
(**A**) Influence of ionic strength on pUC19 condensation (400 ng) by **OC3** and **MC3** (25 μM) opioid compounds. Condensation reactions on pUC19 (400 ng) by opioid compounds in (**B**) acidic NaOAc buffer (80 mM, pH = 4.0), and (**C**) basic Tris buffer (80 mM, pH = 9.0) in the presence of 25 mM NaCl.

Since the biophysical evidence gathered on *C*_3_ opioid–DNA binding points toward a surface-binding coordination mode, we considered the possibility of the tertiary amine within the morphine D (piperidine) ring as a possible cationic site responsible for H-bonding and/or electrostatic binding to the phosphate backbone. Indeed, this particular site is well recognized to undergo protonation prior to binding with specific amino acid residues within opioid receptor cavities (46). To examine this hypothesis, the condensation effects of supercoiled pUC19 were initially examined under sodium chloride titration on agarose electrophoresis. Here, 25 μM aliquots of **MC3**, **OC3** and **HC3** were analyzed with increasing NaCl ionic strength (25–1000 mM) with supercoiled pUC19 (Figure 5A). At low salt concentrations (25–75 mM) the plasmid was condensed by all three opioids, however, in the presence of ≥100 mM NaCl, aggregation by **MC3** and **HC3** agents was inhibited as evidenced by the fraction of DNA migrating in supercoiled form. In contrast, **OC3** maintained its condensation effects up to 750 mM of titrated NaCl and only at the highest ionic strength examined, 1 M, could a fraction of native supercoiled pUC19 be identified. These results indicate that condensation by *C*_3_ morphine analogues are dependent on ionic charge, and that disruption of this interaction by the introduction of an ionic gradient can, at least partially, reverse DNA aggregation. Furthermore, it is worth highlighting that compaction by **MC3** and **HC3** is disrupted to the same degree by NaCl, while **OC3** is substantially more resistant to changes in ionic strength.

To further probe the involvement of quaternary amine cations in the condensation process, aggregation of pUC19 was then examined under acidic (pH 4.0) and basic (pH 9.0) buffered conditions. As anticipated, a clear enhancement of the condensation process at lower pH with sodium acetate buffer (Figure 5B) was observed, while under basic conditions (Tris buffer) DNA aggregation was reduced (Figure 5C). These results indicate that in an acidic environment, the piperidine opioid D ring becomes protonated and collapse of DNA proceeds at lower *C*_3_ concentration. Indeed, on comparing Figure 3A with Figure 5B and C it is clear that condensation in pH 4 buffered solution occurs with the highest efficiency across all three tested opioids, followed thereafter by condensates at pH 7.2 and then finally pH 9.0 where very little aggregation for both **MC3** and **HC3**. This analysis supports proposal that the amine group within the piperidine opioid ring is the protonation site responsible for condensation and, based on these condensation results, is highly probable the 3+ polycationic state of the *C*_3_ symmetric opioid scaffold is required to induce aggregation.

### Atomic force microscopy (AFM) analysis

**Figure 6.**
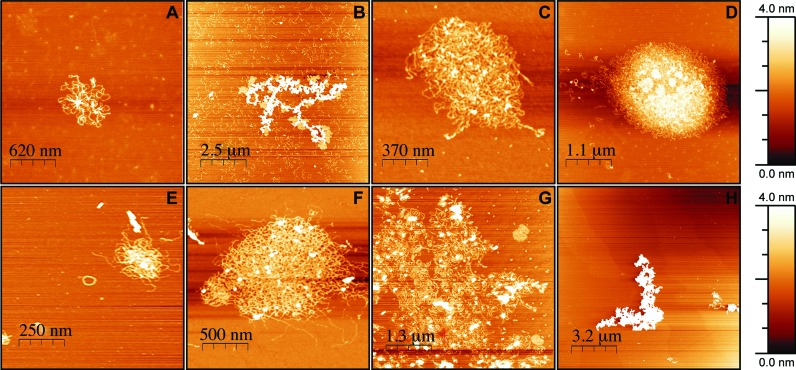
Atomic force microscopy (AFM) images of **MC3**-treated supercoiled and HindIII linearized pUC19 DNA; (**A–D**) supercoiled pUC19 with 8, 9, 10 and 20 μM **MC3**; (**E–H**) linear pUC19 with 5, 10, 20 and 50 μM **MC3**.

AFM imaging provides a direct tool for exploring the effects of ligand binding on DNA morphology (47,48). Due to its nanoscale resolution there are numerous examples of AFM techniques used to determine the binding mode, affinity and site-exclusion number of DNA in the presence of suitable binding substrates. Recent AFM studies have characterized the binding modes of well-studied ligands such as doxorubicin, ethidium bromide and netropsin (49). Moreover, this technique can successfully probe morphological changes induced in closed circular plasmid and linear forms of DNA, along with RNA. In this work, AFM studies were performed with closed circular and HindIII linearized pUC19 in ambient air using a Tapping-Mode with unmodified silicon cantilevers at a 40 N/m force constant. Furthermore, we established that 5 mM of MgCl_2_ was required for complete mica adhesion with no appreciable conformational changes to DNA morphology. **MC3** was selected as a representative compound in this series for AFM analysis. In the presence of 8 μM of **MC3**, supercoiled pUC19 exhibited small cluster formation where tight packing is present in the cluster center with DNA strands extending from its midpoint (Figure 6A). As the concentration of **MC3** increased to 9 μM, a sizeable increase in cluster formation was observed, however, unbound strands of DNA were evident at this point (Figure 6B). A gradual increase in **MC3** concentration to 10 and 20 μM led to the near disappearance of unbound pUC19, leaving only large and tightly packed globules of ∼800 nm and ∼3.3 μm spherical dimension, respectively (Figure 6C and D). Data here supports our earlier gel electrophoresis analysis where 20 μM of **MC3** was required to fully condense the supercoiled plasmid (see Figure 3A). In the presence of linearized pUC19, a lower concentration of **MC3** (5 μM) initiated the onset of condensation (Figure 6E) with both toroidal and cluster formation evident. Toroid condensates, however, were evident at low MC3 concentration only with larger aggregates, >1 μm, appearing at 10 and 20 μM exposure with compact globules forming thereafter (50 μM). These results agree with our electrophoretic data on linear pUC19 (see Figure 3B), where 50 μM of **MC3** was established to condense this sequence.

### Microfluidic analysis of endonuclease inhibition

**Figure 7.**
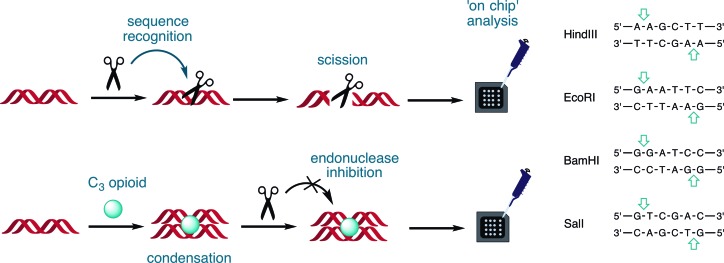
Experimental design for the Bioanalyzer 2100 to identify site-specific endonuclease inhibition by opioid compounds, HindIII, EcoRI, BamHI and SalI.

**Figure 8.**
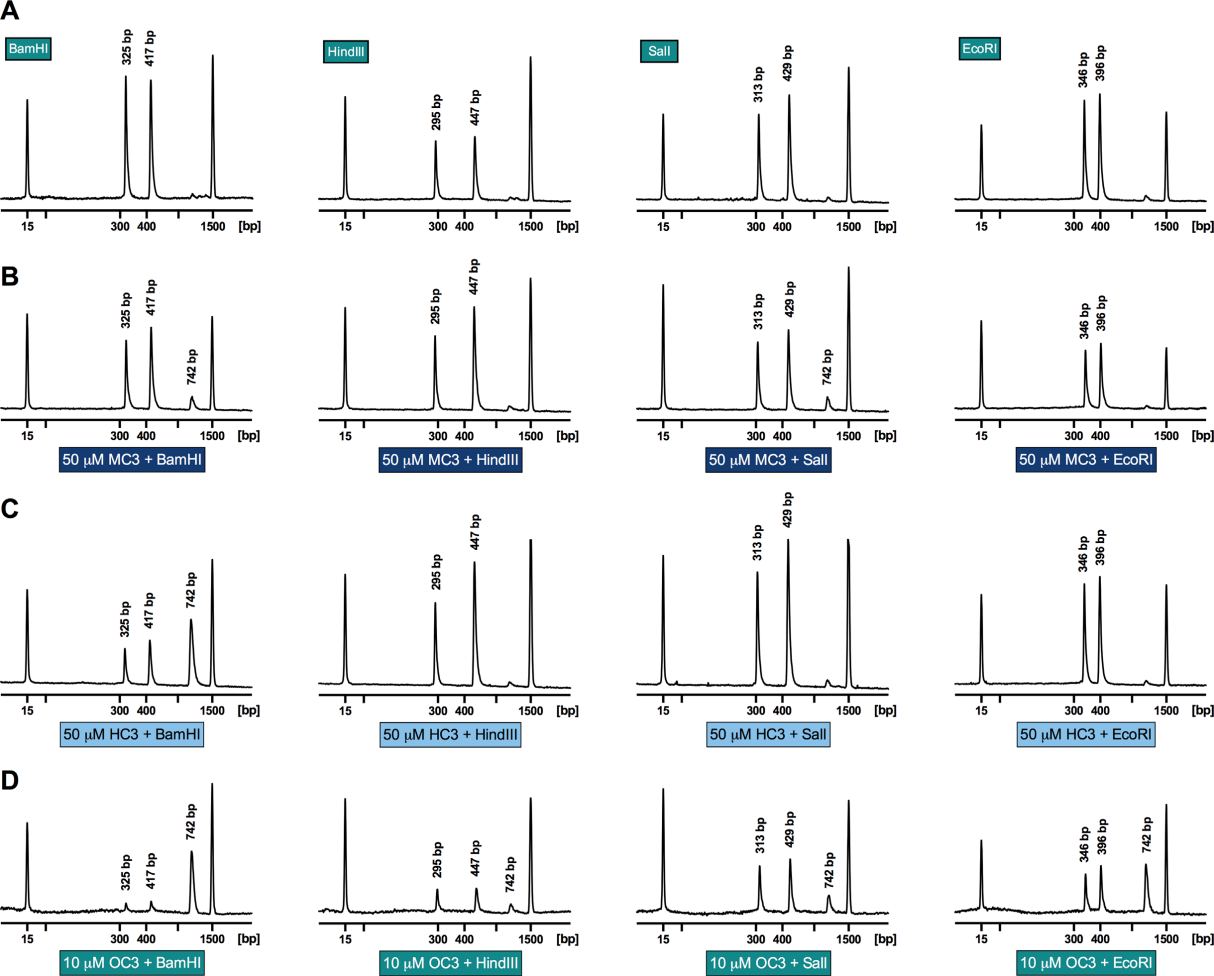
(**A**) Electrograms generated using the Bioanalyzer 2100 of 742 bp dsDNA fragment with treatment by endonucleases BamHI, HindIII, SalI and EcoRI. Electrograms of the 742 bp fragment were pre-incubated for 5 h with either (**B**) **MC3**, (**C**) **HC3** and (**D**) **OC3**, followed by exposure over night to the type II restriction endonuclease.

The condensation effects of the opioid series on a 742 bp dsDNA fragment, amplified from the pUC19 vector encompassing the lacZα gene (Supplementary Section S-6), and subsequent access by sequence-recognition type II restriction endonucleases were initially examined by gel electrophoresis. Results showed the inhibition of migration at ∼20 μM of *C*_3_ opioid exposure indicating condensation had occurred. An on-chip microfluidic assay using an Aglient 2100 Bioanalyzer was then employed (Figure 7) to determine whether *C*_3_-opoid induced condensation could block sequence recognition by type II restriction enzymes BamHI, HindIII, SalI and EcoRI. These restriction enzymes were selected based on their single recognition site within our transcript with control experiments (Supplementary Figure 17) establishing no direct interaction between restriction enzymes and *C*_3_ opiates. A DNA 1000 microfluidic chip was employed in this study to detect and quantify excision fragments, resulting in the disappearance of the parent 742 bp band and the emergence of daughter fragments sized between 295 and 447 bp (Figure 8A). Pre-incubation of the transcript with 50 μM of both **MC3** and **HC3** afforded little protection toward endonuclease accessibility (Figure 8B and C). An exception, however, was noted for BamHI activity to **HC3** exposed DNA; here, the parent fragment at 742 bp emerged with notable reduction in the excision fragment peak area at 325 and 417 bp. Interestingly, pre-incubation of the transcript with low micromolar loading of **OC3** (10 μM) was found to inhibit endonuclease accessibility by BamHI, SalI and EcoRI. Furthermore, in the case of the BamHI-treated transcript pre-exposed to **OC3**, almost complete protection of the oligo was observed. It was noted, however, that the peak area of **OC3** exposed fragments were diminished in comparison to **MC3** and **HC3** experiments, most likely through the enhanced condensation effects of this agent. Finally, in a complementary study using gel electrophoresis, we identified that the non-specific endonuclease DNase I degraded all *C*_3_-opioid plasmid pUC19 condensates with identical efficiency (data not shown).

## CONCLUSION

**Figure 9.**
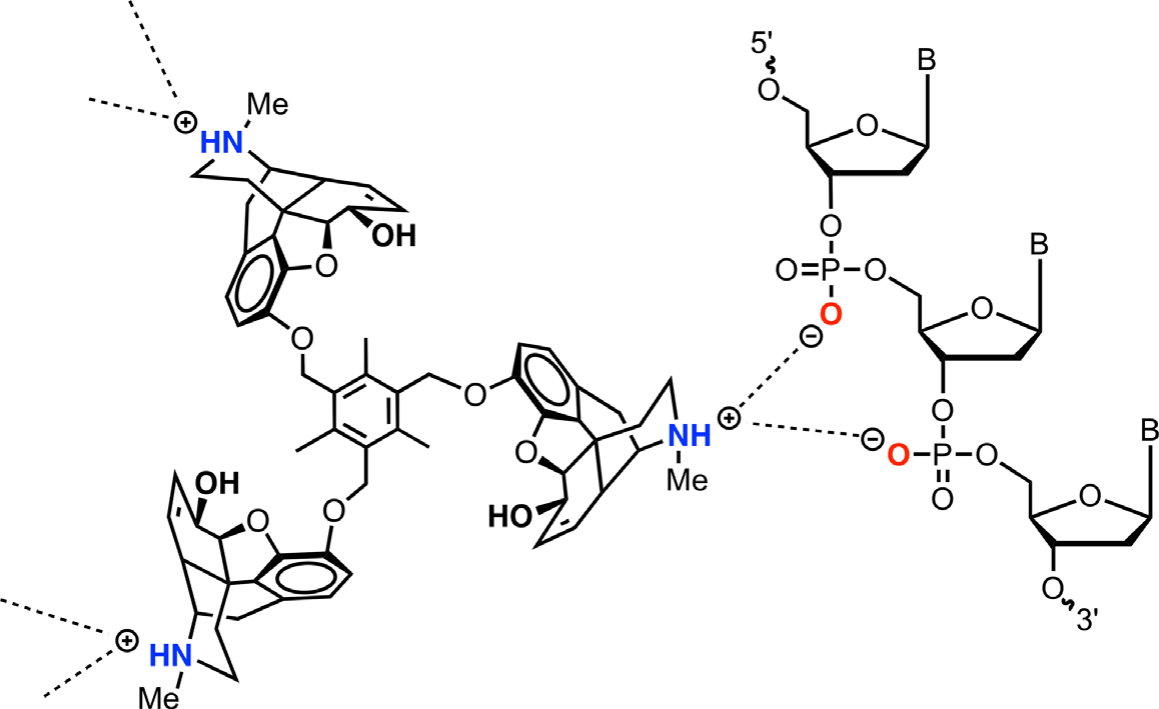
Proposed ionic binding by the *C*_3_ opioid scaffold to the nucleic acid phosphate backbone.

To our knowledge opioid compounds have not previously been shown to interact with nucleic acids. This work may therefore open up new applications for these well-established analgesic compounds as semi-synthetic natural products that facilitate nucleic acid recognition. Target compounds were prepared via short synthetic routes, which in most cases involved a single step (i.e. from an opioid natural product and commercially available benzyl bromide derivative). The high-affinity DNA binding and condensation properties of this scaffold demonstrate the requirement for a *tris*-opiate, along with a polycationic charge of 3+, as the *C*_1_ and *C*_2_ opiate congeners showed negligible condensation effects by comparison. In this regard, this class of agent falls squarely in line with the majority of established DNA condensation agents where a ≥3+ cationic valence requirement for DNA collapse is generally required (22). Biophysical assays on duplex DNA polymers revealed no intercalative or major/minor groove residency, which led to the probing of *C*_3_-opioids as potential electrostatic and/or H-bonding agents. Thus, our preliminary studies using *C*_3_-morphine (**MC3**) focused on the possibility of the 6′-OH, located in the morphine C ring, as a possible surface binding moiety. Further, while physiological conditions promote interplay between morphine protonation (*k*^O^) and zwitterionic (*k*^N^) isomers (Supplementary Section S-8), the 6′-OH does not engage in acid/base speciation and was therefore considered as a potential phosphate-interacting site (50,51). To test this axiom, the development of C3-heterocodeine (**HC3**) and *C*_3_-oripavine (**OC3**) derivatives, where the 6′-OH site is masked by methoxy substituents, was undertaken. Biophysical studies, however, revealed comparable (**HC3**) and enhanced (**OC3**) high-affinity binding to DNA; these data, coupled with condensation properties observed at varying pH and ionic strength, implicate *N*-protonation of the piperidine (E) ring—a known cationic site that undergoes ionic bond formation with the opioid receptor (46)—as the nucleation site responsible for nucleic acid coordination. A proposed model for DNA binding is shown in Figure 9 where each opiate is protonated (and charged) at the piperidine ring that, in turn, surface binds the vicinal nucleic acid backbone. Based on this proposed model, the binding of *C*_3_ opioids to other nucleic acid structures can be expected and preliminary experiments on the condensation of tRNA by **MC3** (Supplementary Figure 38) evidences this. To corroborate the binding mode described in Figure 9, methylation of each *N*-piperidine moiety in **MC3** was achieved (Supplementary Section S-7) leading to isolation of the quateranized piperidium cation **MC3-NMe_2_** as an iodide salt. The condensation effects of **MC3-NMe_2_** with pUC19, when directly compared to **MC3** (Supplementary Figure 16), resulted in a significantly altered condensation profile. Thus, **MC3-NMe_2_** did not aggregate DNA at any exposure value (1–30 μM) where **MC3** exhibited condensation and only at higher concentrations (>30 μM) was **MC3-NMe_2_** condensation observed; we propose this interaction is electrostatic in nature and similar to NMe_2_-quaternarized nucleic acid binding observed in the literature (52,53). It is highly likely, therefore, that *N*-protonation of the *C*_3_ opioid scaffold is responsible for efficient condensation and that piperidium phosphate hydrogen bonding interactions are required for these high-affinity interactions.

**Figure 10.**
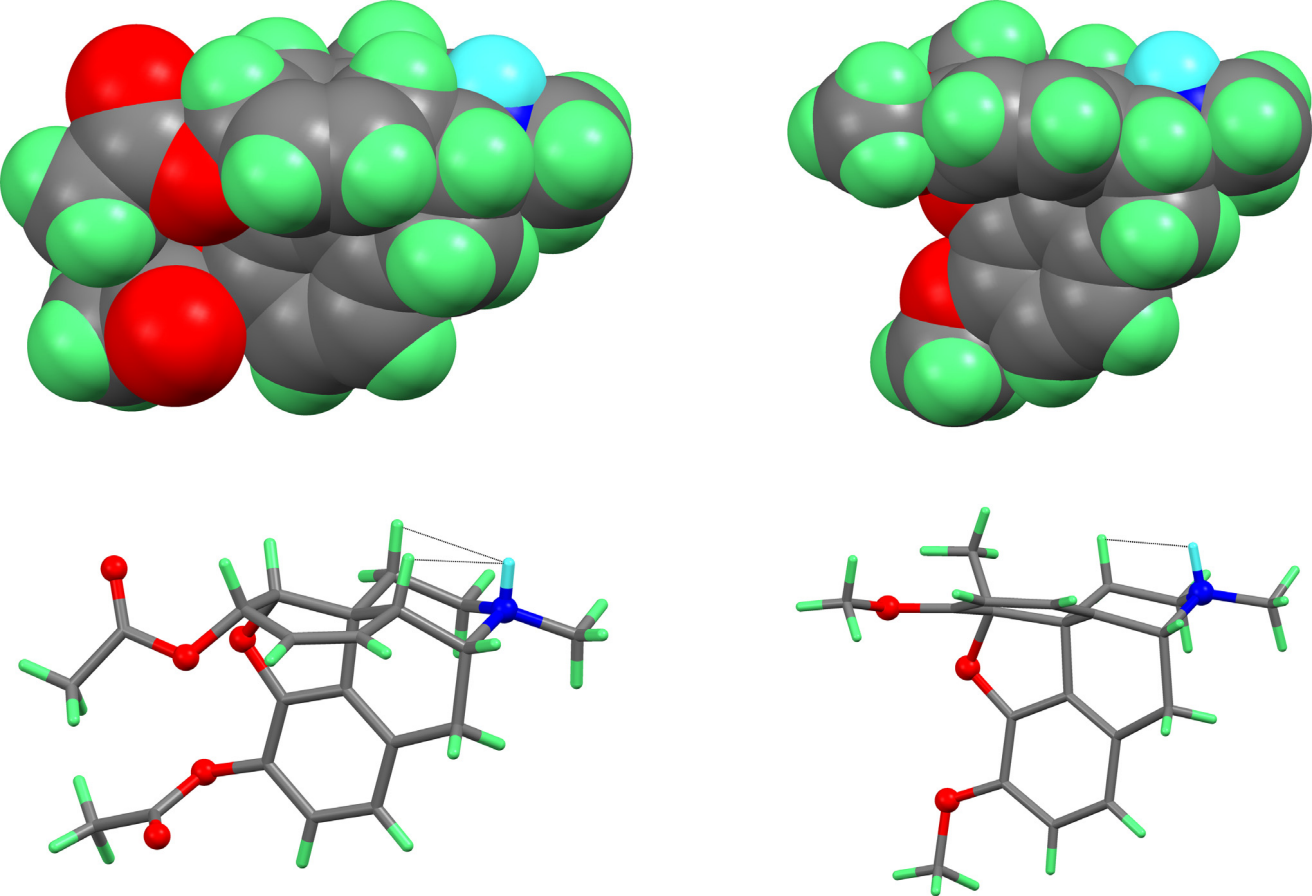
Structures of protonated diacetylmorphine (FAZDAM left) and protonated 3, 6-dimethoxy-5, 17-dimethyl-6,7,8,14-tetradehydro-4,5-epoxymorphinan (LOBGUG right) redrawn from the CSD data (the amine proton has been added to the structure at a calculated position for LOBGUG). The amine proton (highlighted in cyan) is more exposed in the oripavine. Dashed lines show the interactions with neighbouring axial protons.

Further examination of *C*_3_ agents revealed (i) no conformational modification to long canonical DNA polymers, (ii) improved affinity toward aggregation of supercoiled plasmid versus linear DNA conformation and (iii) an overall aggregation activity profile **OC3** >> **HC3** ≈ **MC3**. To derive some insight into the differences in binding between the oripavine compound and the morphine or heterocodeine analogs, we considered structural data for examples of each class taken from the Cambridge Structural Database (CSD) (54). Figure 10 shows the cation of diacetylmorphine hydrogen chloride monohydrate (FAZDAM, ccdc 242245) and 3, 6-dimethoxy-5,17-dimethyl-6,7,8,14-tetradehydro-4,5-epoxymorphinan (LOBGUG, ccdc 985664), for the purpose of comparison a proton has been added to the amine at the calculated position in the latter case. The increased unsaturation in the oripavine means that the ‘C’ ring is flattened relative to the morphine or codeine systems and that one of the hydrogen atoms in an axial position relative to the amine proton is lost. As a consequence, the amine site is clearly less sterically crowded in the oripavine system and this might be expected to make an interaction with the DNA phosphate backbone easier. There are also electronic changes in the π system of ring C, which may influence both the p*K*_a_ of the amine and electronic aspects of bonding.

The condensation process at neutral pH, studied by AFM analysis with **MC3**, identified toroidal formation of pUC19 at low compound loading, while higher loading promoted tightly packed globule formation in both closed circular and linear DNA structures. The combined effect of high binding constants and condensation of the *C*_3_-opioid class was then considered in terms of interrupting protein–DNA recognition (23). Since condensation agents have found widespread utility as transfection agents or carriers of nucleic acids (55,56), protection of this ‘cargo’ from endonuclease degradation is of considerable importance. While DNase I mediated complete transcript degradation from all three *C*_3_-opioid carriers, type II restriction endonucleases inhibition for **HC3** (50 μM) and **OC3** (10 μM) was witnessed at the G–G excision region of BamHI, while **OC3** (10 μM) further protected the G-A excision region of EcoRI. In summary, this work has revealed the discovery of a new high-affinity DNA binding scaffold capable of mediating condensation ostensibly through electrostatic and H-bonding interactions with the phosphate backbone. Our attention now turns to further explore the condensation effects of RNA and alternative DNA structures by these molecules and for their ability to successfully deliver gene vectors.
